# Erythroderma: A Retrospective Study From a Single Institution

**DOI:** 10.7759/cureus.115265

**Published:** 2026-08-26

**Authors:** Kinuko Irie, Yoko Oura, Toshiyuki Yamamoto

**Affiliations:** 1 Dermatology, Fukushima Medical University, Fukushima, JPN

**Keywords:** erythroderma, idiopathic erythroderma, il-17, il-25, tarc

## Abstract

Erythroderma is a severe, chronic condition that can affect more than 90% of the body surface area, occurring either idiopathically or secondarily to various underlying diseases. There have been several reports of erythroderma from outside Japan, while reports from inside Japan are limited. In this study, we retrospectively reviewed the clinical characteristics, underlying diseases, and treatments of patients diagnosed with erythroderma at our department between 2008 and 2024. A total of 147 patients were identified (122 males, 25 females; mean age, 69.2 years). Idiopathic erythroderma was the most common etiology (n = 50, 34.0%), followed by chronic eczema (*n* = 30, 20.4%), psoriasis (*n* = 22, 15.0%), drug eruptions (*n* = 19, 12.9%), malignancies (*n* = 14, 9.5%), and bullous diseases (*n* = 9, 6.1%). Malignancies included both hematologic malignancies (*n* = 3, 2.0%) and solid cancers (*n* = 11, 7.5%). Serum levels of thymus and activation-regulated chemokine (TARC) and IL-25 were significantly elevated in patients with erythroderma, supporting the notion that erythroderma favors Th2-dominant conditions. In addition, erythroderma can occur in association with malignancies. Cases without underlying inflammatory conditions should be screened for internal malignancies, and even if none are detected, careful follow-up is necessary.

## Introduction

Erythroderma is characterized by widespread confluent erythema accompanied by pruritus [1]. Secondary erythroderma may arise from a variety of underlying diseases, including eczema, psoriasis, atopic dermatitis, drug eruptions, lymphoma, lichen planus, pityriasis rubra pilaris, and autoimmune bullous diseases. In immunosuppressed patients, crusted scabies should also be considered [1]. Erythroderma can be classified into three major types: (1) cases in which the primary disease, such as atopic dermatitis, psoriasis, or autoimmune bullous disorders, can be readily identified; (2) cases in which the etiology, such as drug eruption or lymphoma, is revealed after repeated investigations; and (3) cases in which the cause remains unknown despite extensive workup [1]. While diagnosis is straightforward based on clinical features, identifying the underlying causes, triggers, and comorbid conditions is essential after the initial diagnosis. Evaluation should include a detailed medication history, lymph node examination, blood tests, skin biopsy, and malignancy screening [1,2]. Since erythroderma may represent the non-bullous stage of bullous pemphigoid, it is advisable to store skin and serum samples. The frequency of associated malignancies varies across reports [3-7]. Additionally, although several regional studies have been reported from Japan, nationwide comprehensive data remain limited. Laboratory findings often include elevated serum immunoglobulin E (IgE) and thymus and activation-regulated chemokine (TARC) levels and peripheral eosinophilia, similar to those in atopic dermatitis, indicating a Th2 cytokine imbalance as a pathogenic factor1. Although erythroderma appears to be a Th2-shifted disease, the complete pathogenesis remains obscure. Interleukin (IL)-25, also known as IL-17E, has been suggested to be a Th2 cytokine that plays a role in allergic inflammation [3,4]. In the present study, we retrospectively examined the clinical characteristics, underlying diseases, and treatments of erythroderma. In addition, serum IL-25 levels were measured in patients with idiopathic, psoriatic, and atopic erythroderma.

## Materials and methods

The study included patients diagnosed with erythroderma at our department between January 2008 and December 2024. Diagnosis was based on clinical findings of erythema affecting more than 90% of the body surface area.

Patients with insufficient clinical data were excluded from the analysis. Erythroderma was classified as idiopathic or secondary according to the presence or absence of an identifiable underlying disease based on clinical, histopathological, and laboratory findings.

We retrospectively collected data from medical records, including results of skin biopsy, blood tests, infection screening, and CT-based malignancy screening.

In addition, serum samples were collected from patients with idiopathic erythroderma (*n* = 20, 35.1%), psoriatic erythroderma (*n* = 13, 22.8%), atopic erythroderma (*n* = 11, 19.3%), and healthy controls (*n* = 13, 22.8%). Sera were stored at -80 °C, and IL-25 concentrations were measured using a commercially available enzyme-linked immunosorbent assay kit (Mybiosource Inc., San Diego, CA).

Continuous variables are presented as the median (interquartile range (IQR)). Statistical analyses were performed using appropriate tests, including the Steel-Dwass test for multiple comparisons. A *P*-value <0.05 was considered statistically significant.

Due to the retrospective nature of the study, selection bias could not be completely excluded. This study was approved by the Research Ethics Committee of Fukushima Medical University (No. 2025-0320).

## Results

A total of 147 patients were identified: 122 (83.0%) males and 25 (17.0%) females (M:F, 4.9:1), aged 19-92 years (median age, 71 years; IQR, 63-78) (Figure 1). 

**Figure 1:**
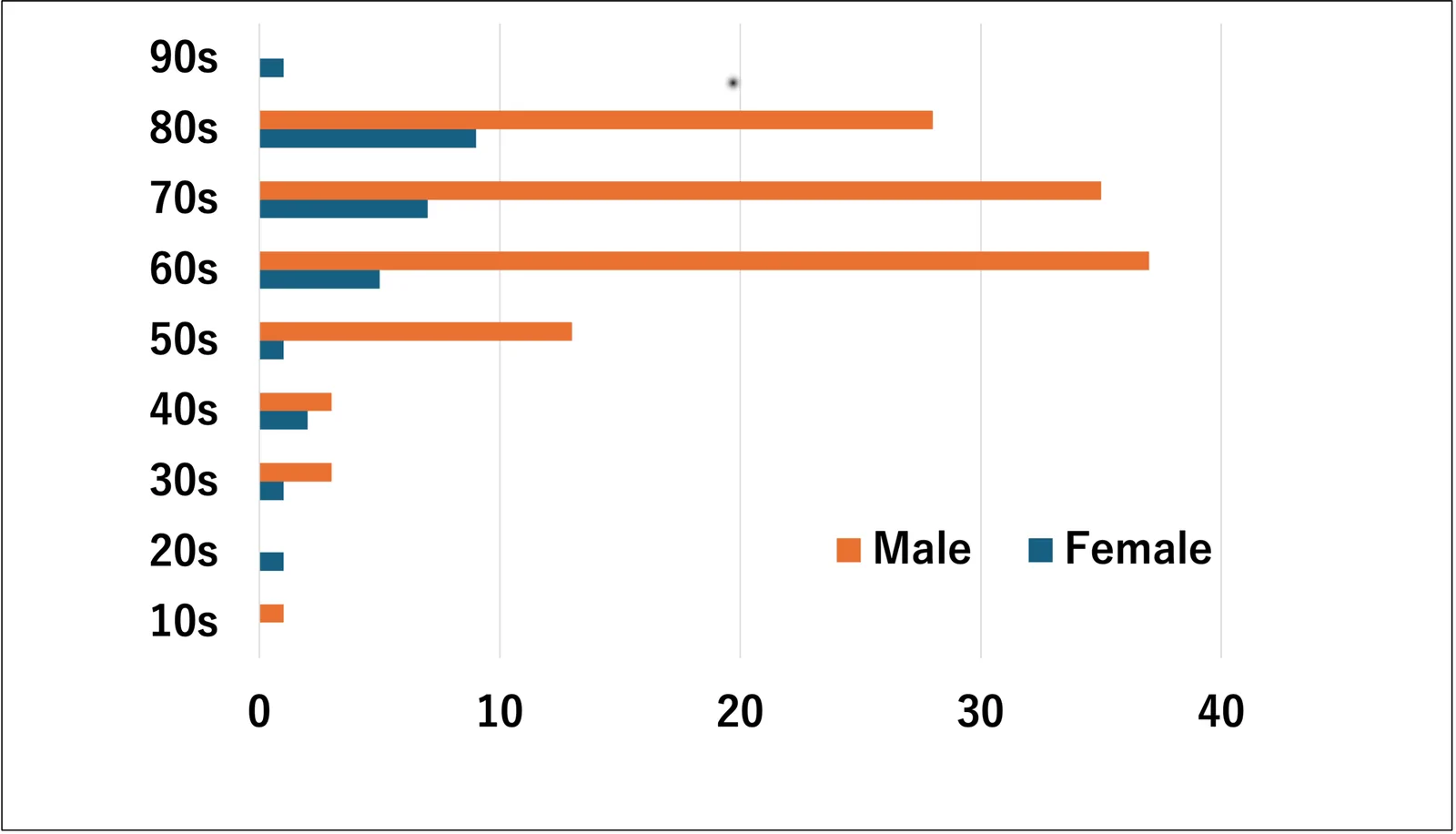
Age distribution of erythroderma by age group.

Pruritus was present in almost all patients at diagnosis, and fever occurred in 26 (17.7%) cases. The distribution of underlying diseases is shown in Table 1.

**Table 1:** Underlying diseases associated with erythroderma.

Underlying diseases	Patients (M:F)	%
Idiopathic	50 (41:9)	34
Chronic eczema	30 (25:5)	20.4
Psoriasis	22 (20:2)	15
Drug eruption	19 (15:4)	12.9
Malignancy	14 (12:2)	9.5
Bullous disease	9 (6:3)	6.1
Metal allergy	1 (1:0)	0.7
Ichthyosis	1 (1:0)	0.7
Scabies	1 (1:0)	0.7

Idiopathic erythroderma was defined as cases in which no identifiable underlying cause was detected through laboratory testing and imaging studies during approximately six months of follow-up. Idiopathic erythroderma accounted for the largest proportion (*n* = 50, 34%), followed by chronic eczema (*n* = 30, 20.4%). In this study, the term *metal allergy* was used to describe allergic contact dermatitis associated with metal exposure, diagnosed on the basis of clinical findings and confirmed by patch testing when available. The frequency of underlying diseases differed between sexes (Figure 2).

**Figure 2:**
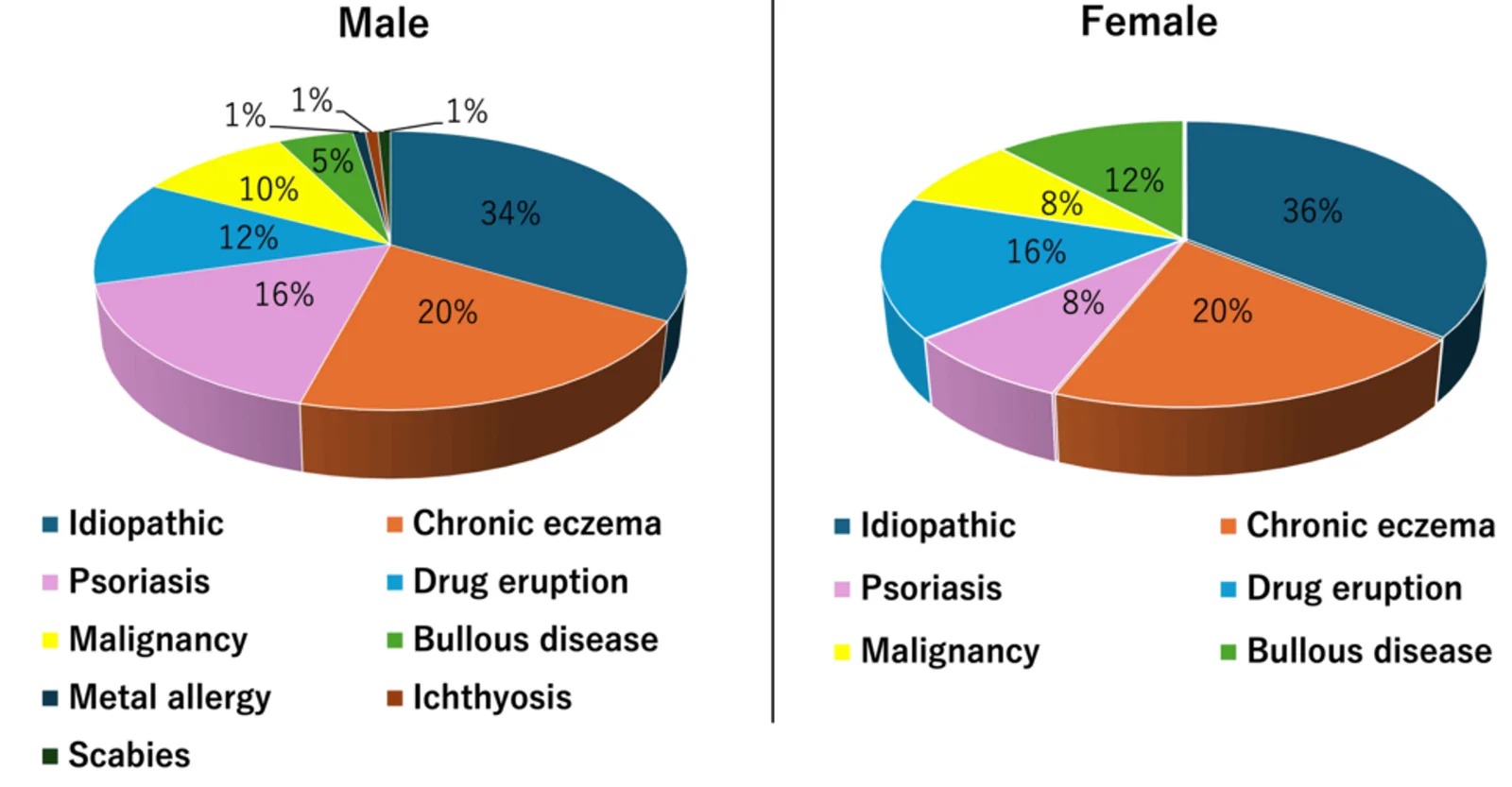
Distribution of erythroderma etiologies by sex.

Concomitant malignancy was observed in 14 patients (9.5%), including hematologic malignancies (*n* = 3, 2.0%) and solid cancers (*n* = 11, 7.5%). Hematologic malignancies included cutaneous T-cell lymphoma (*n* = 2, 1.4%) and adult T-cell leukemia/lymphoma (*n* = 1, 0.7%). Solid tumors included lung cancer (*n* = 3, 2.0%), gastric cancer (*n* = 2, 1.4%), cholangiocarcinoma (*n* = 1, 0.7%), prostate cancer (*n* = 1, 0.7%), esophageal cancer (*n* = 1, 0.7%), rectal cancer (*n* = 1, 0.7%), oropharyngeal cancer (*n* = 1, 0.7%), and hepatocellular carcinoma (*n* = 1, 0.7%) (Table 2).

**Table 2:** Types of associated malignancies.

Patient	Malignancy	Sex	Age (years)
A	Mycosis fungoides	M	66
B	Rectal cancer	M	63
C	Mycosis fungoides	M	32
D	Cholangiocarcinoma	M	89
E	Lung cancer	M	73
F	Lung cancer	M	66
G	Oropharyngeal cancer	M	66
H	Gastric cancer	M	69
I	Prostate cancer	M	78
J	Lung cancer	M	66
K	Esophageal cancer	F	79
L	Hepatocellular carcinoma	M	83
M	Gastric cancer	M	69
N	Adult T-cell leukemia/lymphoma (ATLL)	F	63

Two cases of CTCL, two cases of lung cancer, and one case of prostate cancer were newly diagnosed during the evaluation of erythroderma, whereas the other malignancies were pre-existing conditions. In a subset of patients, malignancies were detected at an advanced stage, precluding surgical intervention and necessitating palliative management. Figure 3 shows representative clinical images of erythroderma.

**Figure 3:**
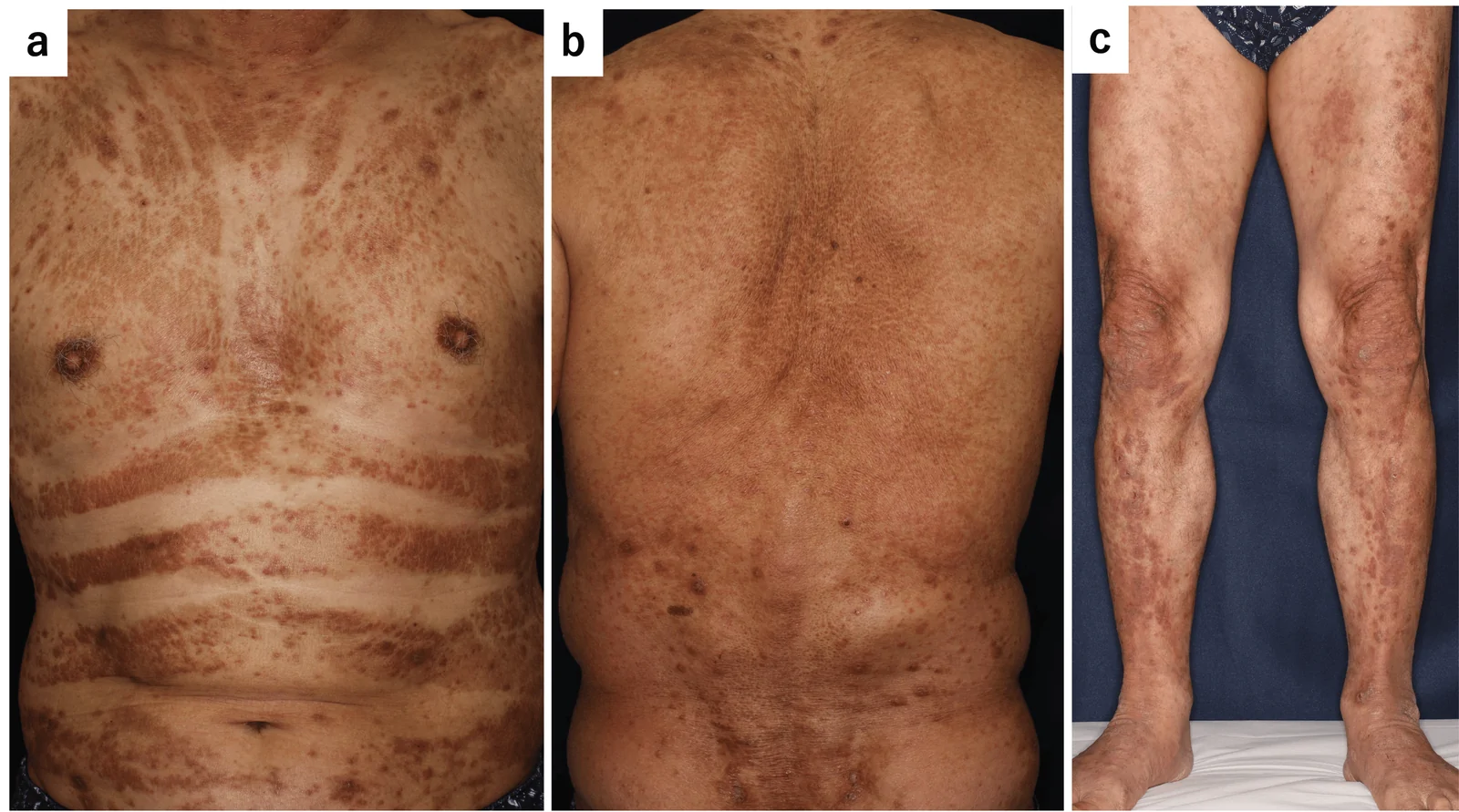
(a-c) Representative clinical photograph of erythroderma associated with gastric cancer.

One patient with concomitant gastric cancer exhibited extensive erythema on the trunk, showing a deck-chair sign. In another patient, early gastric cancer was detected during erythroderma workup, and endoscopic submucosal dissection was performed five months after the first dermatology visit; improvement in erythroderma was observed after 18 months’ follow-up (Figure 4).

**Figure 4:**
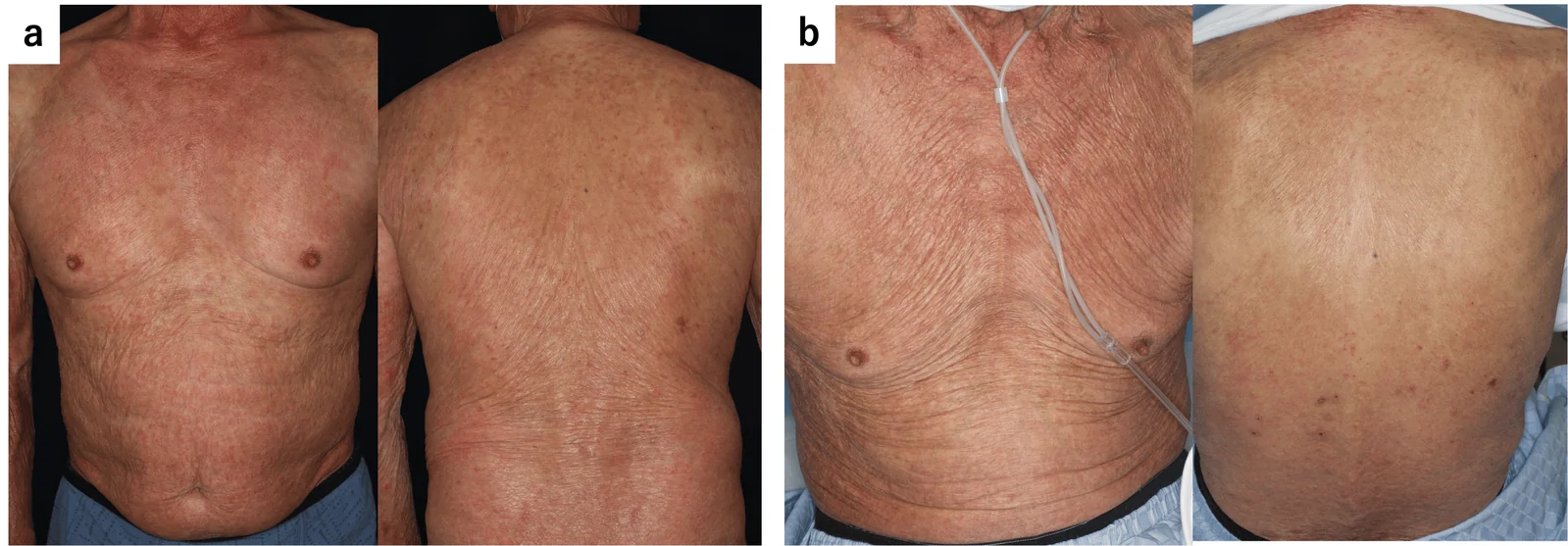
Clinical appearance of erythroderma before and after surgery for malignancy at: (a) initial presentation; (b) 18 months after surgery.

Treatment was tailored to the underlying disease. Therapeutic interventions, including overlaps, were as follows: topical corticosteroids (*n* = 137, 93.2%), oral corticosteroids (*n* = 79, 53.7%), phototherapy (*n* = 21, 14.3%), oral cyclosporine (*n* = 15, 10.2%), oral etretinate (*n* = 7, 4.8%), biologics (*n* = 2, 1.4%), intravenous immunoglobulin (*n* = 1, 0.7%), and plasma exchange (*n* = 1, 0.7%). Idiopathic erythroderma was primarily treated with topical corticosteroids. A comparison between idiopathic and secondary erythroderma revealed no significant differences in age or sex distribution (Table 3).

**Table 3:** Comparison between idiopathic and secondary erythroderma in our series.

Parameter	Idiopathic	Secondary
Age range (years)	44-92	19-89
Mean age (years)	70.5	68.6
Male:Female (N)	41:9	81:16
Mean sIL-2R (U/mL))	2,600	2,113
Mean TARC (pg/mL)	24,702	17,614
Mean LDH (U/L)	409	324
Mean IgE (IU/mL)	6,684	6,989
Mean eosinophil percentage	15%	11%

No significant differences were observed in serum sIL-2R, TARC, or IgE between the two groups. Serum LDH levels were significantly higher in patients with idiopathic erythroderma than in those with secondary erythroderma (*P* = 0.003). Although serum TARC levels tended to be higher in idiopathic erythroderma (*P* = 0.092), the proportion of patients with elevated TARC levels did not differ significantly between the two groups (27/34, 79.4%, vs. 50/67, 74.6%, *P* = 0.805). Serum IL-25 levels were significantly elevated in patients with idiopathic erythroderma (589.8 ± 76.0 pg/mL), psoriatic erythroderma (115.1 ± 28.7 pg/mL), and atopic erythroderma (534.6 ± 106.4 pg/mL) compared with healthy controls (25.5 ± 10.5 pg/mL) (Steel-Dwass test) (Figure 5).

**Figure 5:**
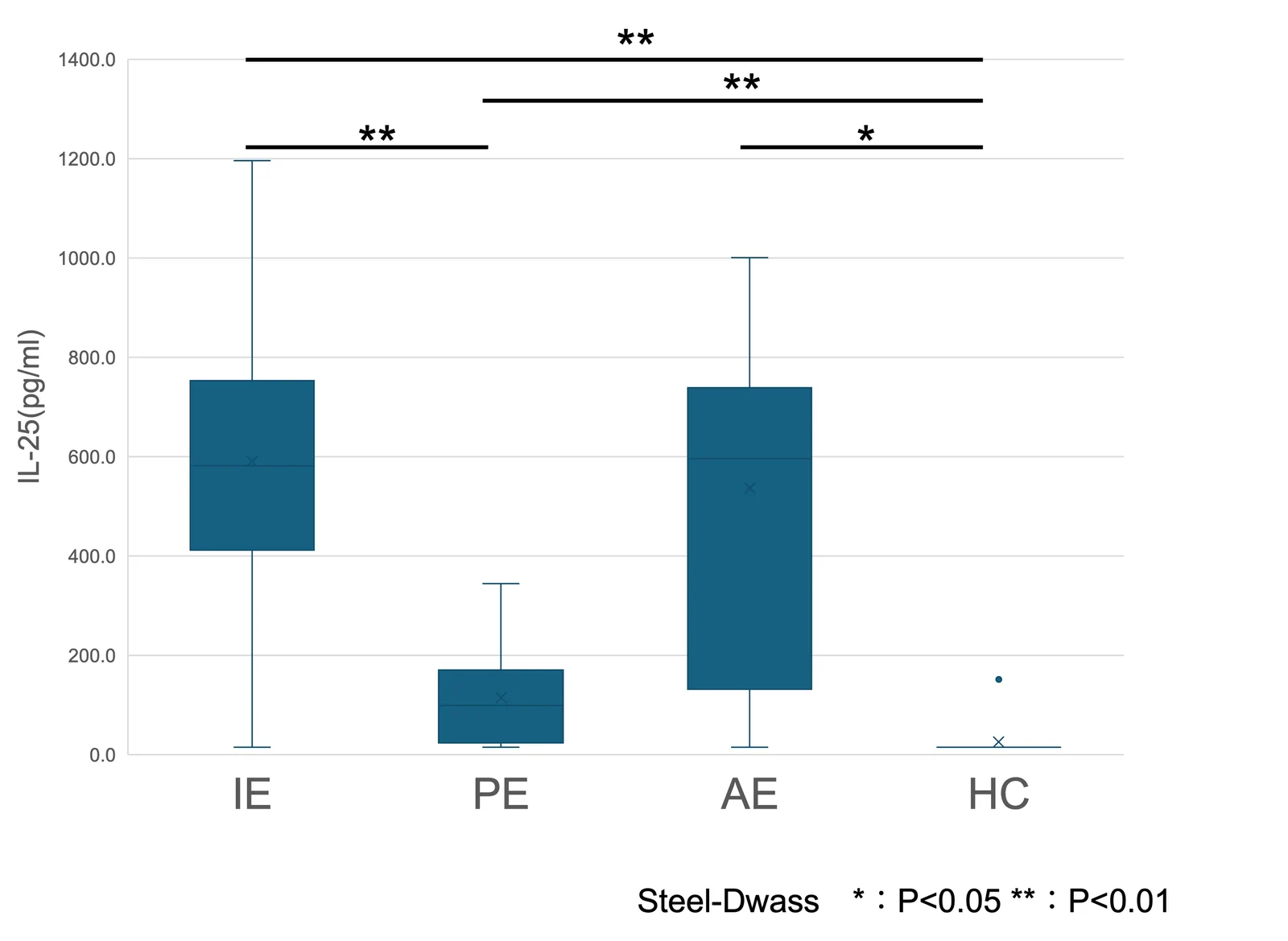
Serum IL-25 levels in patients with idiopathic erythroderma (IE), psoriatic erythroderma (PE), atopic erythroderma (AE), and healthy controls (HC). *P < 0.05. **P < 0.01 (Steel-Dwass test).

## Discussion

Comparison of the present study with previous studies [2,5-18] revealed marked variability in the reported proportions of idiopathic erythroderma (0-47.1%), drug-induced erythroderma (1.5%-46.6%), and malignancy-associated erythroderma (0-17.8%) (Table 4).

**Table 4:** Comparison between our series and previously published reports since 2000.

Reference	N	Idiopathic (%)	Preexisting dermatoses (%)	Drug (%)	Malignancy (%)
Sehgal et al. (2004) [5]	80	22	58	20	0
Akhyani et al. (2005) [6]	97	7.2	57.9	21.6	10.3
Rym et al. (2005) [7]	80	7.5	72.5	11.3	8.8
Yuan et al. (2010) [8]	82	6.1	68.3	17	4.9
Khaled et al. (2010) [9]	82	25.6	43.9	21.9	4.87
Li et al. (2012) [2]	260	14.2	70.8	12.7	2.3
Tan et al. (2014) [10]	225	14.2	68.9	10.7	4
Nakano‐Tahara et al.(2015) [11]	68	47.1	39.7	1.5	8.8
César et al. (2016) [12]	103	3.9	65	18.4	11.7
Mathew et al. (2017) [13]	370	15.7	74.6	6.5	3.2
Sheikh et al. (2019) [14]	92	0	43.5	38	17.4
Miyashiro et al. (2020) [15]	309	16.8	46.2	12.3	17.8
Long et al. (2023) [16]	74	ND	85.1	4.1	5.4
Kondo et al. (2024) [17]	88	2.3	48.9	46.6	2.3
Kliniec et al. (2024) [18]	212	19.1	37.2	10.8	13.2
Present study	147	34	42.2	12.9	9.5

These differences may be attributed to variations in patient populations and study settings. As a tertiary referral center, our institution may receive a higher proportion of severe or refractory cases, as well as patients with suspected or confirmed malignancy, which could influence the observed distribution of etiologies. In addition, differences in diagnostic strategies and the extent of systemic evaluation may affect the detection of underlying diseases. Furthermore, the definition of idiopathic erythroderma varies across studies, particularly with respect to the duration and thoroughness of follow-up; cases initially classified as idiopathic may later be reclassified when an underlying disease becomes apparent. These factors should be considered when interpreting the relatively high proportion of idiopathic cases in our cohort. 

To date, several case-series reports on erythroderma have been published from single institutions or multicenter studies [2,10-16]. Although the etiologies of erythroderma are diverse, the majority of cases develop secondary to pre-existing dermatoses, with eczema, psoriasis, atopic dermatitis, drug eruptions, and CTCL being the most common. The reported frequency of drug-induced erythroderma varies widely, ranging from 1.5% to 46.6%. Erythroderma may also present as a paraneoplastic manifestation of malignancies, including internal solid tumors and hematologic malignancies, which account for 0-17.8% of erythroderma cases. Hematologic malignancies include mycosis fungoides, Sézary syndrome, CTCL, adult T-cell leukemia/lymphoma, and Hodgkin lymphoma [19]. In this study, the incidence of malignancy-associated erythroderma was 9.5%. The proportion of idiopathic erythroderma, in which no underlying disease could be identified, has been reported to range from 0% to 47.1%. Since erythroderma can manifest as a cutaneous sign of malignancy, systemic evaluation is essential, as highlighted by the detection of malignancies in our cohort.

Pathogenetically, elevated serum TARC and IgE levels, as well as peripheral eosinophilia, are frequently observed in erythroderma, suggesting a Th2-skewed immune response [11]. In our series, elevated TARC levels tended to be more frequent in idiopathic erythroderma than in secondary erythroderma. IL-25 induces the expression of Th2-dominant cytokines [3,4] and has been reported to be upregulated in psoriatic erythroderma [20]. However, its role across different types of erythroderma remains unclear. In the present study, serum IL-25 levels were elevated regardless of the underlying disease, suggesting a possible association between IL-25 and erythrodermic conditions. These findings may indicate a shift toward Th2-related immune responses; however, the results should be interpreted with caution, as this study is observational and does not establish a causal relationship. Recent studies have expanded our understanding of disease mechanisms, highlighting the roles of IL-31 in mediating pruritus and chronic inflammation [21], cytotoxic CD8⁺KLRK1⁺ T cells in idiopathic erythroderma [19], and Mas-related G-protein coupled receptor member X2 (MRGPRX2) in neuro-immune crosstalk [22]. MRGPRX2, which is highly expressed on mast cells, has also been implicated in various pruritic dermatologic conditions. These findings provide novel insights into the biological basis of the disease and identify potential therapeutic targets that may extend beyond conventional immunosuppressive therapies. In chronic cases, histiocytes are also observed in skin biopsy specimens. In both idiopathic and secondary erythroderma, infiltration of CD68-positive and CD163-positive M2 macrophages has been reported, suggesting a possible involvement of M2 macrophages in the pathogenesis of these conditions [22]. Furthermore, elevated levels of IL-17 have been observed in a subset of erythroderma cases, including psoriatic erythroderma [23,24]. Further studies are warranted to elucidate the roles of Th17 cells and type 2 innate lymphoid cells in the pathogenesis of erythroderma.

Age-related dysregulation of immune system function in humans, referred to as immunosenescence, is influenced by factors such as innate immune responses, genetic susceptibility, and the gut and skin microbiota. Immunosenescence is characterized by an increase in proinflammatory cytokines and a reduction in the anti-inflammatory milieu, ultimately leading to a state of low-grade chronic inflammation known as inflammaging [25,26]. Inflammaging is associated with elevated levels of proinflammatory cytokines, including TNF-α, IL-1α/β, IL-6, IL-8, and IFN-γ.

In addition, skin aging induces xerosis. Disruption of the epidermal barrier enhances the production of proinflammatory cytokines and promotes cutaneous inflammation. A Th2-skewed immune response with aging has been reported in mice [27]; however, whether a similar phenomenon occurs in humans remains unclear. Furthermore, age-related Th17/Treg imbalance [28] and a decline in Th17 cell numbers [29] have also been documented.

Therapeutic approaches should be tailored to the underlying disease. While topical corticosteroids remain the mainstay of treatment, they are often insufficient in widespread erythroderma. Oral corticosteroids may provide rapid symptomatic control, but their long-term use is limited by adverse effects and the risk of relapse. Cyclosporine can be effective, particularly in inflammatory erythroderma, although careful monitoring for renal dysfunction and hypertension is required. Phototherapy can be beneficial in some patients, although its application may be difficult in severe or unstable cases [2,10-15]. However, refractory cases remain challenging. Recent therapeutic advances for atopic dermatitis, including biologic agents and Janus kinase (JAK) inhibitors, have revolutionized the management of severe cases [4]. Applying these therapies to erythroderma, especially when the underlying etiology is identified, is promising in terms of efficacy and disease management. Since IL-4/IL-13 pathways are implicated in Th2-dominant erythroderma [30], biologics targeting these cytokines may represent a rational and potentially effective treatment option in selected patients.

There are several limitations in this study. This study was conducted at a single tertiary referral center, which may have introduced selection bias and referral bias. More severe or atypical cases of erythroderma are more likely to be referred to our institution, potentially limiting the generalizability of the findings to community-based settings. Consequently, the clinical distribution and cytokine profiles observed in this cohort may not fully represent the broader erythroderma population. In addition, because this was a retrospective study, follow-up duration varied among patients, and some patients were lost to follow-up, making comprehensive assessment of long-term clinical outcomes and treatment responses difficult. Furthermore, the cytokine analysis was conducted in a relatively small number of patients, and serum cytokine levels were measured at a single time point without longitudinal evaluation. Previous treatments may also have influenced serum cytokine levels. Accordingly, the observed elevation of IL-25 should be interpreted as an association with erythroderma rather than evidence of a causal role in its pathogenesis.

## Conclusions

In conclusion, erythroderma represents a heterogeneous clinical entity with diverse etiologies and complex immunological backgrounds. Our single-center retrospective analysis highlights the predominance of idiopathic cases and suggests an association between Th2-associated cytokines, including IL-25, and erythroderma.
